# Transcriptomic and Metabolomic Analyses Reveal Differences in Flavonoid Pathway Gene Expression Profiles between Two *Dendrobium* Varieties during Vernalization

**DOI:** 10.3390/ijms241311039

**Published:** 2023-07-03

**Authors:** Wenbo Shu, Meirong Shi, Qiqi Zhang, Wenyu Xie, Liwei Chu, Mingxuan Qiu, Linyan Li, Zhixin Zeng, Lei Han, Zhenyuan Sun

**Affiliations:** 1 National Key Laboratory for Germplasm Innovation and Utilization Crops, College of Horticulture and Forestry, Huazhong Agriculture University, Wuhan 430070, China; wenboshu@mail.hzau.edu.cn (W.S.);; 2 Key Laboratory of Tree Breeding and Cultivation of the State Forestry Administration, Research Institute of Forestry, Chinese Academy of Forestry, Beijing 100091, China; meirongshi99@yeah.net (M.S.);

**Keywords:** *Dendrobium capillipes*, *Dendrobium nobile*, vernalization, transcriptome, metabolome, flavonoid biosynthesis, flavone and flavonol biosynthesis

## Abstract

*Dendrobium* (Orchidaceae, Epidendoideae) plants have flowers with a wide variety of colors that persist for a long period throughout the year. The yellow coloration of *Dendrobium* flowers is mainly determined by the flavonol pathway and the flavone pathway, but the relevant biosynthesis mechanisms during vernalization remain unclear. To explore the similarities and differences in flavonoid biosynthesis in different tissues during vernalization, we selected two species of *Dendrobium* for a flower color study: *Dendrobium capillipes* Rchb (which has yellow flowers) and *Dendrobium nobile* Lindl (which has white flowers). We collected a total of 36 samples from six tissue types and both *Dendrobium* species during vernalization and subjected the samples to metabolic profiling and transcriptome sequencing. A total of 31,504 differentially expressed genes (DEGs) were identified between different tissues of the two *Dendrobium* species by transcriptomic analysis. However, many differentially accumulated metabolites (DAMs) and DEGs were enriched not only in the general pathway of “flavonoid biosynthesis” but also in multiple subpathways of “flavone and flavonol biosynthesis”. According to a combined transcriptome and metabolome analysis, Putrescine hydroxycinnamoyl transferase 1 (LOC110093422) may be the main gene responsible for the differences in flavonoid accumulation during vernalization, which is closely associated with yellow flowers. Taken together, the results of our study preliminarily revealed the metabolites responsible for and the key genes regulating flavonoid biosynthesis during vernalization. These results provide a basis for the further study of the molecular mechanism of flavonoid synthesis during vernalization.

## 1. Introduction

The genus *Dendrobium* is a member of the largest family of flowering plants and accounts for 20% of total orchid sales; the flowers of these plants have a wide variety of colors and persist for a long time throughout the year [1]. These plants fall into two categories, including phalaenopsis-type and nobile-type plants, and include more than 1500 species and artificial hybrids of high medicinal and ornamental value distributed across tropical and subtropical Asia and Oceanica [2]. Among these species, more than 80 are native to China [3] and are mainly distributed south of the Qinling Mountains [4].

Due to the commercial value of these species as ornamental plants, an increasing number of studies have been reported on *Dendrobium* spp. in recent years [5]. The flower colors of *Dendrobium* include dark red, pink, purple, yellow, white, green, etc. The factors that determine flower colors include temperature, metal ions, pH, flavonoid copigments, secondary metabolites, and the morphology of epidermal cells [6]. To meet consumer demands, the continual development of novel flower colors is expected. However, the development of new varieties of *Dendrobium* with different flower colors is limited by the long breeding time required. Given these limitations, effective approaches for the manipulation of *Dendrobium* flower color are needed. Flower color is an important trait of many plants, and flower coloration has been among the popular research topics of biological studies [7]. *Dendrobium* flowers contain flavonoids, polysaccharides, alkaloids, phenanthrene, bibenzyl, and sesquiterpenes, as well as other active compounds [8]. Flavonoids are a class of widely distributed secondary metabolites that are synthesized through the phenylpropanoid metabolic pathway, in which phenylalanine is converted to naringenin, which then enters the anthocyanin, flavone, and flavonol biosynthesis pathways [2,9]. The accumulation of flavonoids also has an age-dependent effect, but there are remarkable differences between different tissues [8]. *Dendrobium* flower bud differentiation, which can be divided into seven phases [10], can be completed in 56 days at 22/17 °C, or in 35 days at 18/13 °C [10,11,12,13,14].

There are six main groups of flavonoids in plant tissues: anthocyanins, flavan-3-ols, flavanonols, flavones, flavonols, and phenolic acids [7]. The yellow coloration of *Dendrobium* flowers is mainly determined by the flavone and flavonol pathways [8]. Many genes involved in flavonoid biosynthesis have also been characterized in *Dendrobium*. However, additional studies are still needed to determine several key factors involved in the regulation of yellow flowers [5]. Metabolomic and transcriptomic techniques are efficient approaches for elucidating the regulatory networks of flower color in *Dendrobium* [2]. Gene coexpression analysis has been used to discover new candidate genes, identify key modulators, and elucidate regulatory pathways [4]. However, these methods are hindered by the lack of cultivars with different phenotypic traits, and few studies have reported on whether the distribution and metabolism of flavonoids is related to flower color during *Dendrobium* vernalization. To date, the accumulation mechanisms of the main flavonoids in the different organs of *Dendrobium* remain elusive during vernalization, and the regulatory mechanisms of flavonoids in *Dendrobium* are still unclear. Therefore, it is necessary to compare flavonoid biosynthesis pathways between different *Dendrobium* tissues during vernalization, which will be helpful for a comprehensive evaluation of the relationship between flavonoid contents and flower colors.

**Figure 1:**
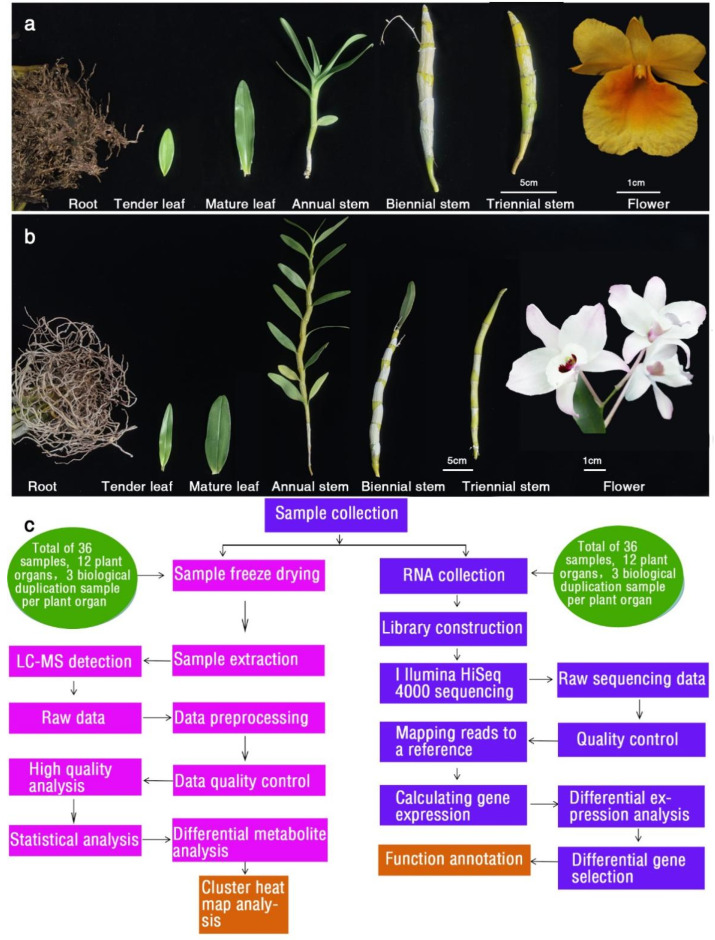
Study workflow from sample preparation through sample processing and analysis. (**a**) Six different tissues of *D. capillipes* used for metabolite and transcriptome sequencing analysis. (**b**) Six different tissues of *D. nobile* used for metabolite and transcriptome sequencing analysis. (**c**) Schematic overview of the study.

In this study, integrated metabolomic and transcriptomic analyses were applied to elucidate the molecular mechanisms underlying the flower coloration of a novel yellow-flowered variety of *D. capillipes* and a white–purple-flowered variety of *D. nobile* (Figure 1a,b) during vernalization. This study evaluated the similarity and specificity of the flavonoid biosynthesis pathway in different *Dendrobium* tissues to discern the differences in flower color and provide theoretical support for the selection of the vernalization period.

## 2. Results

### 2.1. General Description of the RNA Sequencing Data

To explore the molecular mechanism related to the yellow coloration of *Dendrobium* petals, we performed RNA-Seq using samples collected from root, young leaf, mature leaf, annual stem, biennial stem, and triennial stem tissues of *D. nobile* and *D. capillipes* (Figure 1). After the removal of low-quality sequencing data, a total of 250.65 gigabases (Gb) of transcriptomic data were obtained from the collected samples (no less than 5.8 Gb per sample). The experimental processes involved in the study are shown in Figure 1c.

**Figure 2:**
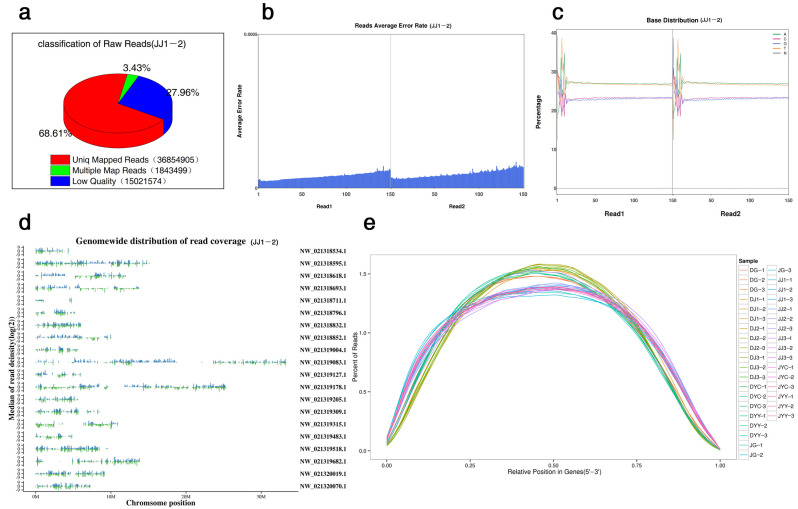
Global assessment of transcriptomic data. Examples of results for the (**a**) sequencing error rate distribution, (**b**) A/T/G/C content distribution, (**c**) composition of raw data, and (**d**) location and coverage depth distribution of mapped reads in the reference genome, which reflect the good quality of the transcriptomic libraries. (**e**) Location map of reads mapped on mRNAs: the abscissa is the normalized mRNA location, and the ordinate is the percentage of reads in the total mapped reads in the corresponding location interval. The figure reflects all mRNA summary of mapped reads proportions within the interval.

The initial sequence obtained after sequencing was used for a subsequent analysis after a series of quality control steps (Figure 2a). First, the distribution of the sequencing error rate was evaluated (Figure 2b). The error rate of each sequenced base was determined according to the Phred score (Q_phred_) by the formula (1: Q_phred_ = −10 log_10_(e)), and the Phred value was subjected to a base-calling analysis. The probability model showed that this model can accurately predict the error rate of base discrimination. Subsequently, the distribution of the A/T/G/C content was investigated to determine the presence or absence of AT and CG separation (Figure 2c). In addition, the clean data obtained by filtering the raw data and the clean reads were compared to the chromosome data (Figure 2d). The raw count was homogenized for all samples by the log_2_ (count +1) method, and distribution density statistics were calculated (Figure 2e).

### 2.2. Functional Enrichment Analysis of DEGs

**Figure 3:**
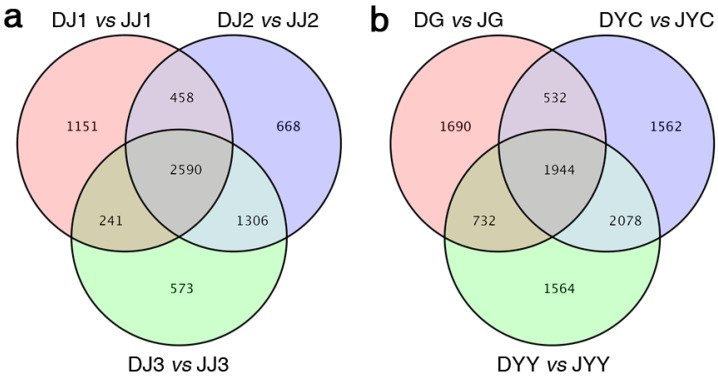
Venn diagram showing differentially expressed genes in the same stem organs of the two species (**a**) and in the same root, young leaf, and mature leaf organs of the two species (**b**). *D. capillipes* tissues (root (DG), young leaf (DYY), mature leaf (DYC), annual stem (DJ1), biennial stem (DJ2) and triennial stem (DJ3) tissues), and *D. nobile* tissues (root (JG), young leaf (JYY), mature leaf (JYC), annual stem (JJ1), biennial stem (JJ2), and triennial stem (JJ3) tissues).

To identify DEGs, genes with an adjusted *p* value <0.01, as determined by DESeq2, were considered differentially expressed. The DEGs in different tissues were subsequently screened. The spatiotemporal expression differences among the genes are presented in the form of a Venn diagram (Figure 3a,b). A total of 31,504 DEGs were identified in different comparisons between both species. Furthermore, 4898, 4440, 5022, 4710, 6116, and 6318 DEGs were identified in the pairwise comparisons of DG vs. JG, DJ1 vs. JJ1, DJ2 vs. JJ2, DJ3 vs. JJ3, DYC vs. JYC, and DYY vs. JYY, respectively. At different levels, approximately 241 to 2590 differentially expressed genes were screened in the different tissues (Figure 3a,b). Accordingly, hierarchical clustering analysis also showed a clear difference in the expression patterns of the DEGs between different tissues (Figure 3). Interestingly, the number of DEGs between DJ2 and JJ2 was much greater than that between the same tissues at other ages (Figure 3a,b). However, a Venn diagram illustrated that 668 genes were unique to stem tissue at this age (DJ2 vs. JJ2) (Figure 3). The levels of these shared DEGs differed significantly from DJ2 vs. JJ2 in the stems at other ages during vernalization in the two species and may be related to the yellow color.

### 2.3. KEGG Pathway Enrichment Analyses of DEGs

**Figure 4:**
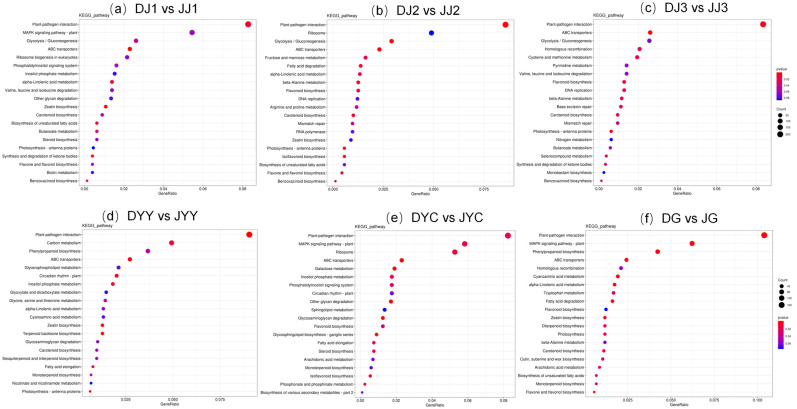
KEGG enrichment analysis of DEGs in the different tissues of *D. capillipes* and *D. nobile*. The ordinate indicates the pathway name, and the abscissa is the enrichment factor. The size of the circle indicates the number of genes enriched in the KEGG pathway, and the color of the circle represents the *p* value.

To further reveal the potential roles of the DEGs in biological functions, KEGG enrichment analysis of DEGs was performed for comparisons of DG vs. JG, DJ1 vs. JJ1, DJ2 vs. JJ2, DJ3 vs. JJ3, DYC vs. JYC, and DYY vs. JYY, which was plotted as a scatter diagram (Figure 4). The degree of KEGG enrichment was determined based on the Q-value, rich factor, and the number of genes in a given pathway. The results revealed that DEGs were mainly enriched in the categories of plant–pathogen interaction pathways, ABC transporters, ribosomes, fatty acid degradation, flavonoid biosynthesis, carotenoid biosynthesis, and flavone and flavonol pathways. For each comparison, the 20 most significantly enriched pathway entries are shown (Figure 4), or all entries are shown if less than 20 pathway entries were enriched. The results showed that the pathways associated with flavonoid biosynthesis and flavone and flavonol were enriched. In particular, flavonoid biosynthesis pathways and flavone and flavonol pathways were all significantly enhanced for DJ2 vs. JJ2 and DG vs. JG, “flavonoid biosynthesis” was enriched in DJ3 vs. JJ3 and DYC vs. JYC (Figure 4), and “flavone and flavonol biosynthesis” was enriched in DJ1 vs. JJ1 (Figure 4, Table S2). Flavonoid biosynthesis pathways and flavone and flavonol pathways were detected in DJ2 vs. JJ2, and we hypothesize that DEGs in these two metabolic pathways have important effects on the formation of the yellow color.

### 2.4. Analysis of DEGs Related to the Flavonoid Biosynthesis Pathway

**Figure 5:**
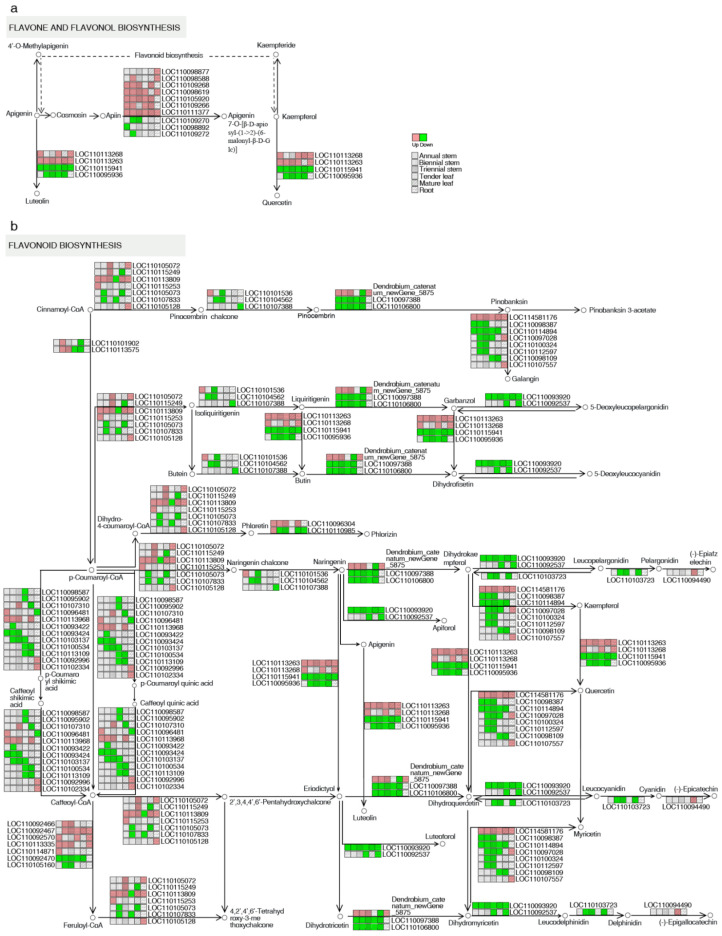
Schematic overview of the major flavonoid biosynthesis pathway of different genes in the tissues of *D. capillipes* and *D. nobile*. The flavone and flavonol biosynthesis pathway (**a**) and the flavonoid biosynthesis pathway (**b**) are shown.

The biosynthesis pathway of flavonoids, which are secondary plant metabolites, has been thoroughly studied in many other plant species. By comparing *Dendrobium* plants with yellow- and white-colored flowers, we observed extensively downregulated or upregulated genes involved in the biosynthesis of flavones, flavonols, and flavonoids in yellow flowers relative to white flowers. Moreover, many transcription factors, including p-coumaroyl-CoA, naringenin chalcone, naringenin, dihydrokae-mpferol, caffeoyl-CoA, caffeoyl quinic acid, apigenin, eriodictyol, dihydromyricetin, kaempferol, and leucocyanidin, were predicted to regulate anthocyanin biosynthesis-related structural genes, and these genes might play crucial roles in the biosynthesis of flavonoids in *Dendrobium* (Figure 5). Collectively, the results of this study provide further information for the identification of candidate genes for the functional characterization and manipulation of flower color in *Dendrobium*.

Based on the comparison of different tissues between the two *Dendrobium* species, we conducted a detailed analysis of the genes involved in the flavonoid, flavonol, and flavone biosynthesis pathways. A total of 18 DEGs potentially involved in flavonol and flavone biosynthesis were identified, including apigenin, apigenin, and kaempferol. In total, 46 DEGs potentially related to flavonoid biosynthesis were found, including cinnamoyl-CoA, pinocembrin chalcone, pinocembrin, pinobanksin, pinobanksin 3-acetate, isoliquiritigenin, liquiritigenin, garbanzol, buterin, butin, dihydrofisetin, p-coumaroyl-CoA, dihydro-4-coumaroyl-CoA, phloretin, naringenin chalcone, naringenin, dihydrokae-mpferol, leucopelargonidin, pelargonidin, caffeoyl shikimic acid, caffeoyl-CoA, caffeoyl quinic acid, apigenin, eriodictyol, dihydromyricetin, kaempferol, leucocyanidin, cyanidin, feruloyl-CoA, dihydromyricetin, leucodelphinidin, and delphinidin (Figure 5 and Table S1). Genes encoding flavonoid 3′-monooxygenase (LOC110113263), isoflavone 7-O-glucoside-6″-O-malonyltransferase (LOC110111377), and flavonol synthase 6 (LOC114581176) were upregulated in all five groups, and those encoding flavonoid 3′-monooxygenase (LOC110115941) and dihydroflavonol 4-reductase/flavanone 4-reductase (LOC110093920) were downregulated in all five groups. The gene encoding putrescine hydroxycinnamoyl transferase 1 (LOC110093422) was only downregulated in 2-year-old stems among all five groups. Genes encoding isoflavone 7-O-glucoside-6″-O-malonyltransferase (LOC110098588) and trans-cinnamate 4-monooxygenase (LOC110101902) were only upregulated in 2-year-old stems among the three stem tissues, and those encoding isoflavone 7-O-glucoside-6″-O-malonyltransferase (LOC110109272), chalcone synthase (LOC110105073), and chalcone synthase 8 (LOC110107833) were only downregulated in 2-year-old stems among the three stem tissues (Table 1).

### 2.5. Validation of DEGs by qRT–PCR

To further confirm the accuracy of the transcriptomic data, RNA-Seq analysis and reverse-transcription PCR (qRT–PCR) were performed on randomly selected DEGs to determine the authenticity and reliability of the transcriptome data and differential expression of the candidate genes. We selected 12 differentially expressed genes (LOC110113263, LOC110111377, LOC110098588, LOC110109272, LOC110105073, LOC110097028, LOC110107388, LOC110113109, LOC114581176, LOC110093422, LOC110115941, and LOC110093920) of the flavonoid biosynthesis pathway for qRT–PCR verification. Although there were slight differences between the results of RNA-Seq and qRT–PCR, the overall trend was basically consistent (Figure S1). Hence, the transcriptome sequencing was reliable.

### 2.6. Differential Metabolite (DAM) Analysis in Different Tissues during Vernalization

**Figure 6:**
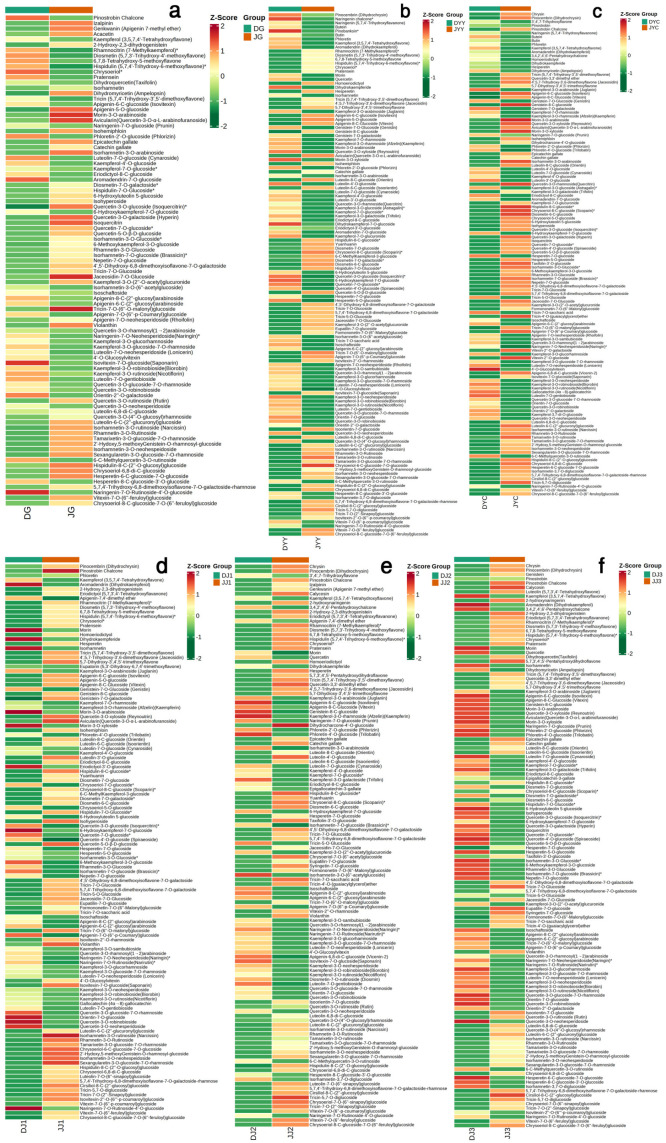
Variation in flavonoid content in different tissues of different species. The “flavonoid biosynthesis” and “flavone and flavonol biosynthesis” of DAM in the DG vs. JG (**a**), DYY vs. JYY (**b**), DYC vs. JYC (**c**), DJ1 vs. JJI (**d**), DJ2 vs. JJ2 (**e**), and DJ3 vs. JJ3 (**f**) comparison groups. Each column represents a *Dendrobium* sample, and each row represents a critical metabolite. The mean data were normalized via logarithmic transformations and displayed on a color scale where red, black, and green indicate high, intermediate, and low compound concentration values, respectively. * means the substance has an isomer.

To further explore the secondary metabolic mechanism of the different tissues between the two *Dendrobium* species, we used the targeted metabolomics approach and investigated the changes in the main metabolites between the different flowers of the two *Dendrobium* species with the UPLCMS/MS system (Figure 6), aiming to provide a more comprehensive landscape of the metabolites involved during vernalization in the two *Dendrobium* species. We first performed a KEGG enrichment analysis of DAMs in G, YY, YC, J1, J2, and J3 tissues (Figure S2) to identify the main relevant metabolic pathways. The results revealed that, in the six comparison groups, the “biosynthesis of secondary metabolites” pathway was the key pathway in the KEGG enrichment analysis. Interestingly, the “flavonoid biosynthesis” and “flavone and flavonol biosynthesis” pathways were highly enriched in the DG vs. JG, DYY vs. JYY, DYC vs. JYC, DJ1 vs. JJI, DJ2 vs. JJ2, and DJ3 vs. JJ3 comparison groups (Figure S2). The results of the metabolome analysis showed that differential flavonoid metabolites accounted for the most significant proportion of the total differential metabolites, with many differential substances identified. Few studies have qualitatively studied flavonoid metabolites during the vernalization of *Dendrobium*. To further understand the molecular mechanisms of flavonoids, a metabolomic analysis of different tissues was performed. The metabolomic profiling results showed that there were marked differences in many flavonoids in the six organs between the two different *Dendrobium* species (Figure 6, Table S3). In DG vs. JG, 91 DAMs belonging to the flavonoid pathway were identified with 38 increased and 53 decreased metabolites. In DYY vs. JYY, 131 DAMs belonging to the flavonoid pathway were identified with 64 increased and 67 decreased metabolites. In DYC vs. JYC, 128 DAMs belonging to the flavonoid pathway were identified with 49 increased and 79 decreased metabolites. In DJ1 vs. JJI, 122 DAMs belonging to the flavonoid pathway were identified with 41 increased and 81 decreased metabolites. In DJ2 vs. JJ2, 124 DAMs belonging to the flavonoid pathway were identified with 53 increased and 71 decreased metabolites. In DJ3 vs. JJ3, 130 DAMs belonging to the flavonoid pathway were identified with 66 increased and 64 decreased metabolites (Figure 6, Table S3). More metabolites were downregulated than upregulated, indicating that relatively few metabolites efficiently accumulated in the different tissues (Figure 6, Table S3).

### 2.7. Integrated Analysis of Transcriptomics and Metabolomics

In this study, transcriptome and metabolome data were integrated to thoroughly elucidate the mechanisms involved in the different tissues examined between two *Dendrobium* species during vernalization. The KEGG enrichment analysis of DAMs and DEGs (Figure 4 and Figure S2) demonstrated that 14, 16, 20, 20, 17, and 19 pathways were simultaneously enriched in DG vs. JG, DYY vs. JYY, DYC vs. JYC, DJ1 vs. JJI, DJ2 vs. JJ2, and DJ3 vs. JJ3, respectively. Notably, the “flavonoid biosynthesis” and “flavone and flavonol biosynthesis” pathways, in particular, were significantly enriched in almost all of the comparison groups, indicating that the two pathways played a crucial role in the two *Dendrobium* species during vernalization. We conducted a comprehensive analysis of transcript and metabolite levels associated with the flavonoid biosynthesis pathway in two *Dendrobium* species to better understand the relationship between genes and metabolites. It was also found that the expression levels of genes related to metabolites in the different tissues of *Dendrobium* were not completely consistent (Figure 5 and Figure 6), suggesting that genes involved in substance synthesis are regulated by a complex network. However, all comparison groups showed variation in many genes, and their downstream metabolites in the flavonoid biosynthesis pathway were also generally consistent (Figure 5 and Figure 6), as found for p-coumaroyl-CoA and LOC110093422, naringenin and LOC110113263, apigenin and LOC110113263, and kaempferol and LOC110113263. This suggests that flavonoid synthesis genes may be critical during vernalization.

## 3. Discussion

Metabolomics is a histological technology that emerged after proteomics and genomics [15]. Based on high-throughput methods, many metabolites can be simultaneously analyzed by metabolomics, and the corresponding relationships between metabolites and physiological changes can be elucidated [16]. Therefore, it can be used to detect changes in metabolites in different plant tissues. However, only the *D. officinale* genome has been released, while *D. capillipes* and *D. nobile* reference genomes are not available. We previously performed de novo transcriptome analysis of data from *D. capillipes* and *D. nobile* (Figure 2), and a total of 4898, 4440, 5022, 4710, 6116, and 6318 DEGs were identified in the pairwise comparison of DG vs. JG, DJ1 vs. JJ1, DJ2 vs. JJ2, DJ3 vs. JJ3, DYC vs. JYC, and DYY vs. JYY, respectively (Figure 3a,b). Integrated analysis of the transcriptome and metabolome has been applied to reveal the regulatory pathways of specific agronomic traits [8]. In this study, the metabolomes and transcriptomes of six different tissues of two *Dendrobium* species during vernalization were analyzed. Metabolomics analysis revealed differentially accumulated metabolites during vernalization. Integrated data analysis showed that hundreds of metabolites and thousands of genes were differentially expressed. Previous studies have shown that the *D. officinale* genome can be used to analyze the transcriptome data of *D. huoshanense* and *D. moniliforme* with a high mapping rate [8]. Similar results were obtained in this study. Therefore, it is feasible to use *D. officinale* as a reference sequence for a comparative transcriptome analysis of the two *Dendrobium* plants.

In general, high temperature represses, while low temperature promotes, the biosynthesis of flavonoids [17]. The KEGG enrichment analysis of DAMs and DEGs (Figure 4 and Figure S2) demonstrated many differential metabolites involved in the “flavonoid biosynthesis” and “flavone and flavonol biosynthesis” pathways in all of the comparison groups. Notably, the expression of flavonoid biosynthesis-related genes was highly correlated with flavonoid metabolite abundance during vernalization (Figure 5 and Figure 6). Flavonoids are an important class of secondary metabolites in plants and are widely distributed in the leaves, stems, and roots of *Dendrobium* [16]. It was found that different growth and development stages of plants, including different seasons and ages, are important factors affecting the accumulation of secondary metabolites in plants [18]. Flavonoids are the main active ingredients of *Dendrobium*, and their accumulation patterns vary at different developmental stages and at different ages of plants [19]. According to their structural differences, flavonoids can be mainly divided into flavones, flavonols, flavandiols, chalcones, anthocyanins, and proanthocyanidins [20,21], and flavonol synthesis is the causative factor of the yellow coloration [22]. Currently, the biosynthetic pathways of flavonoids have been identified in many plants, but functional gene studies of flavonoid biosynthetic pathways in *Dendrobium* are less frequently reported [20,21]. The flavonoid synthesis process in plants is relatively conserved, most of the enzyme genes involved in the pathway have been cloned, and their functions have been characterized in some model plants [16]. Flavonol biosynthesis is one of the important branches of the flavonoid biosynthetic pathway [23]. However, there are few studies on the biosynthetic metabolic pathways of *Dendrobium* flavonoids [16]. Therefore, it is likely that these DAMs are the key metabolites underlying the dynamic variations in flavonoids, and these genes play a crucial role in the synthetic pathway of flavonoids during vernalization.

Flavonoids are an important class of secondary metabolites of plants that fulfil a variety of functions in plants [24]. Some studies have revealed the roles of flavonoids in regulating the activities of a wide range of proteins and thereby influencing cell growth and differentiation in plants [25]. Flavonoids are widely distributed plant pigments derived from the phenylpropanoid pathway [26]. The flavonoid contents of *D. officinale* largely vary in leaf, stem, and root tissues [20]. Studies have also shown that the primary shade of flower color (from white to yellow) is mainly determined by flavonoid metabolites [27]. In most plants, flavonoids are the main pigments involved in the coloration of organs or tissues. For example, the flower colors of various ornamental plants, such as petunia and chrysanthemum [8], result from tissue-specific flavonoid accumulation. Moreover, different flavonoid components within a plant can also exhibit significant variations in their contents [17]. Flavonoids are synthesized through the phenylpropanoid and polyketide pathways, and the starting point for all flavonoids is malonyl-CoA and p-coumaroyl-CoA [23]. The flavonoid biosynthetic pathway leads to the synthesis of anthocyanins [27], and chalcone synthase is the first key enzyme involved in anthocyanin biosynthesis [26]. The contents of flavonoids in the stems of different *Dendrobium* species showed variations based on metabolic data, and the expression of flavonoid biosynthetic genes can be significantly different in different plant species [8].

Flavonoids are also important in signal transduction between plants [21]. Flavonols, anthocyanins, and procyanidins (PAs) share a common pathway from phenylalanine to dihydroflavonols [28]. Quercetin, isorhamnetin, and kaempferol have also been reported to contribute to the coloration of yellow flowers in *Camellia chrysantha*, *Nelumbo nucifera*, *Lathyrus chrysanthus,* and *Eustoma grandiflorum* [29]. However, gossypetin and quercetin are involved in synthesizing yellow pigment in *P. vulgaris* [22]. *Favanone 3-hydroxylase* (*F3H*) catalyzes the synthesis of *dihydrokaempferol* from naringin (colorless), followed by the synthesis of kaempferol (light yellow) and quercetin (light yellow) mediated by the *flavonol synthase* (*FLS*) [28]. Flavonoids are widely found in Orchidaceae, and flavone C-glycosides and flavonols are the most common components [30]. Flavonoid-related structural genes have been confirmed, but the key enzyme types are not much different among plant species [20]. This finding indicates that the expression of p-Coumaroyl-CoA and LOC110093422 was only upregulated in 2-year-old stems among the three examined stem tissues: naringenin and LOC110113263, apigenin and LOC110113263, and kaempferol and LOC110113263 are correlated, consistent with the results of this study (Figure 5 and Figure 6). Because the expression of DEGs in different tissues is very different (Table 1), it is assumed that flavonoids are influenced by different tissues and are subject to change as plants grow and develop. 

As *Dendrobium* flowers only appear on biennial stems (Table 1), it is likely that LOC110093422 might be closely related to the regulation of key genes that may have something to do with yellow flower formation during vernalization. Spermidine hydroxycinnamoyl transferases might underlie the natural variation in the levels of spermidine conjugates in rice [31]. Spermidine hydroxycinnamoyl transferase is associated with hydroxycinnamoyl-spermine conjugate (HCSpm) accumulation in *S. richardii*, in which HCSpm is instrumental in plant development [32]. But spermidine hydroxycinnamoyl transferase accumulates in the pollen coat in *Arabidopsis thaliana* [33], and *SpmHT* expression is high in flowers in *S. richardii* [32]. In addition, the expression of the *SHT* gene in Fabales affects organ reallocation [33], and spmHT promotes the functional analysis of other genes in the phenylpropanoid pathway [32]. Therefore, LOC110093422 may be related to yellow flower formation during vernalization.

Metabolite and gene expression analyses indicated that metabolites and genes related to flavonoid metabolism play important roles in the different tissues of the two *Dendrobium* species during vernalization. In addition, many key genes (Table 1) that could play a critical role in flavonoid metabolic pathways during vernalization were identified. The objective of this study was to fill the gap in the complete flavonoid biosynthesis pathway for the first time. Compared to traditional breeding strategies, transgenic methods have greater potential for creating novel phenotypes. The introduction of new properties such as new colors in orchids via mutation breeding or crossbreeding is often difficult, but can be achieved relatively easily using transgenic technology. Transgenic research has confirmed that *DseCHS-B* and *DseDFR* impact the flower color of *Dendrobium Sonia* ‘Earsakul’ and that *DOFT* and *DOFTIP1* impact the flowering of *Dendrobium Chao Praya Smile* [17]. However, the functions of these genes and the mechanism of action of transcription factors have not been clarified. We hope that our work has contributed to the understanding of flavonoid biosynthesis in *Dendrobium*.

## 4. Materials and Methods

### 4.1. Overview of Experimental Design and Sample Preparation

*D. capillipes* and *D. nobile* plants were grown in a semiautomatic greenhouse (70% natural light intensity and temperatures ranging from 25 to 28 °C) at the Chinese Academy of Forestry (Beijing, China). On 15 January 2019, three-year-old plants with well-developed pseudobulbs were transferred to a controlled thermoperiodic chamber (RXM Smart climate box (Ningbo, Zhejiang)) regulated at 18 °C (light)/13 °C (dark), with a photoperiod of 12 h (320 µmol m^−1^ s^−1^ *PPF* (13–15 molm^−2^d^−1^ of photosynthetic photons)) provided by fluorescent and incandescent lamps and a mean relative humidity of 70–80% for 30 d [10]. A completely randomized design with 10 single plant replicates for each treatment was used. Plants collected on 15 February 2019 were used as study materials. Six *D. capillipes* tissues (root (DG), young leaf (DYY), mature leaf (DYC), annual stem (DJ1), biennial stem (DJ2), and triennial stem (DJ3) tissues) (Figure 1a), and six *D. nobile* tissues (root (JG), young leaf (JYY), mature leaf (JYC), annual stem (JJ1), biennial stem (JJ2), and triennial stem (JJ3) tissues) (Figure 1b) were manually dissected, flash frozen in liquid nitrogen, and kept at −80 °C. The experimental design and analysis pipeline are shown in Figure 1c. Each tissue was represented by 3 biological replicates, for a total of 36 samples. We selected a total of 30 *Dendrobium* plants (10 *Dendrobium* plants constituted one biological replicate) to collect samples.

### 4.2. Sample Extraction and Analysis of Flavonoid Metabolites

Biological samples were vacuum-freeze-dried via a Scientz-100F instrument. The freeze-dried samples were crushed using a mixing mill (MM 400, Retsch, Shanghai Tusen, Germany) with a zirconia bead, and the extracts were filtered (SCAA-104, 0.22 μm pore size; ANPEL, Shanghai, China, http://www.anpel.com.cn/, accessed on 15 July 2021) before UPLC–MS/MS analysis. The sample extracts were analyzed using a UPLC–ESI–MS/MS system (UPLC, Shimadzu Nexera X2, https://www.shimadzu.com.cn/, accessed on 15 July 2021; MS, Applied Biosystems 4500 QTRAP, https://www.thermofisher.cn/cn/zh/home/brands/applied-biosystems.html, accessed on 15 July 2021). Metabolites that significantly differentially accumulated between groups were determined according to the following criteria: a variable importance in projection (VIP) ≥ 1 and an absolute log_2_ (fold change (FC)) ≥ 1. VIP values were extracted from the orthogonal projections to latent structures–discriminant analysis (OPLS-DA) results, which were also used to generate score plots and permutation plots via the R package MetaboAnalystR. The data were log_2_-transformed and mean-centred before OPLS-DA. Heatmap analysis was applied to visualize the dynamic changes in critical flavonoid metabolites in different tissues of the different species, as shown in Figure 2; each column represents a *Dendrobium* sample, each row represents a critical flavonoid metabolite, and the color-coded scale from green to red corresponds to the content of critical metabolites from low to high, respectively.

### 4.3. RNA Extraction, Library Construction, and RNA Sequencing (RNA-Seq)

We describe how we collected and processed the samples, extracted the mRNA, constructed a cDNA library, controlled the quality of the transcriptomic data, aligned the sequencing data to that of a reference genome (*Dendrobium officinale* Kimura et Migo), and performed a correlation analysis between the samples. Finally, we describe how we obtained reliable data for use in subsequent analysis and future research. Total RNA was extracted from all the samples using a Column Plant RNAout 2.0 kit (Tiandz, Inc., Beijing, China). Quantification and qualification of the RNA concentration and purity were performed using a NanoDrop 2000 instrument (Thermo Fisher Scientific, Wilmington, NC, USA), and the RNA integrity was assessed using the RNA Nano 6000 Assay Kit of the Agilent Bioanalyzer 2100 system (Agilent Technologies, Inc., Santa Clara, CA, USA). Sequencing libraries were generated using an NEBNext Ultra^TM^ RNA Library Prep Kit for Illumina (NEB, MA, USA) following the manufacturer’s recommendations, and index codes were added to attribute sequences to each sample. Clustering of the index-coded samples was performed on a cBot Cluster Generation System using TruSeq PE Cluster Kit v4-cBot-HS (Illumina; NEB, MA, USA) according to the manufacturer’s instructions. After cluster generation, the prepared library was sequenced on an Illumina platform, and paired-end reads were generated. The raw reads were further processed with a bioinformatic pipeline tool, the BMKCloud (www.biocloud.net, OmicShare database) online platform.

### 4.4. Quantification of Gene Transcription Level and Analysis of DEGs

The raw sequences were transformed into clean reads after data processing (Figure 3a). These clean reads were then mapped to the *Dendrobium officinale* reference genome sequence [34]. HISAT2 tools software (Nova-seq 6000) was used to map the reference genome. Gene functions were annotated based on information from the following databases: the NCBI nonredundant protein sequence (Nr), NCBI nonredundant nucleotide sequence (Nt), Protein family (Pfam), Clusters of Orthologous Groups of proteins (KOG/COG), SwissProt (a manually annotated and reviewed protein sequence database), Kyoto Encyclopedia of Genes and Genomes (KEGG) Orthologous (KO), and Gene Ontology (GO) databases. Gene expression levels were estimated according to the fragments per kilobase of transcript per million fragments mapped (FPKM) values. Differential expression analysis of two conditions/groups was performed using DESeq2. Genes with an adjusted *p* value <0.01 according to DESeq2 were considered differentially expressed. Differential expression analysis of two samples was performed using edgeR. A false discovery rate (FDR) <0.01 and fold change ≥2 were set as the thresholds for significantly different expression.

### 4.5. Quantitative Real-Time PCR (qRT–PCR) Validation

Total RNA was extracted from fruit pulp tissue at different developmental stages of the three cultivars using RNAprep Pure (Tiangen, Beijing, China). First-strand cDNA was synthesized using an RNA reverse transcription kit (Toyobo, Shanghai, China). To verify the accuracy of the transcriptome data, we used Primer 5 to design specific primers for 12 genes and performed qRT–PCR analysis (Table S1). These primers were synthesized by Sangon Biotech (Sangon, Shanghai, China). The 2^−ΔΔCT^ method was used for relative quantitative analysis of the data, and the internal reference gene *TATA box binding protein-like* (*TBP*) [35] and SYBR Premix Ex Taq II (Novoprotein, Shanghai, China) were employed to validate the DEG expression results. Three biological and three technical replicates of each reaction were analyzed on a LightCycler^®^ 480 instrument (Roche, Switzerland) with a first step of 95 °C for 5 min for pre-denaturation, followed by 40 cycles of 95 °C for 10 s for denaturation, and 60 °C for 30 s for annealing/extension [35]. All primers are listed in Table S1.

### 4.6. Statistical Analysis

Statistical analysis was performed using SPSS Statistics 19.0 software (IBM, Armonk, NY, USA). One-way ANOVA and Duncan’s multiple comparison test were used to analyze the differences between samples of the different tissues during vernalization.

### 4.7. Data Records

A detailed tutorial of the processing steps performed in R and the complete code used for data processing are provided in the Supplementary Materials. The FASTQ files for the raw RNA-Seq reads in this study have been deposited in the NCBI Sequence Read Archive (SRA) under the study accession PRJNA806377 (https://dataview.ncbi.nlm.nih.gov/object/PRJNA806377?reviewer=rj3do6ltpu9gvltc6f18u0jdpf, https://www.ncbi.nlm.nih.gov/sra/, accessed on 15 July 2021) [36].

## 5. Conclusions

This study combined transcriptomic and metabolomic analyses to explore the mechanisms underlying the differential expression of flavonoid biosynthesis genes in G, YY, YC, J1, J2, and J3 tissues. We identified eleven potential DEGs encoding key enzymes and reconstructed the flavonoid biosynthetic pathway contributing to the yellow flower coloration of *Dendrobium*. In addition, DEGs related to anthocyanin biosynthesis were detected. Among these DEGs, *putrescine hydroxycinnamoyl transferase 1* (LOC110093422) represents a key candidate gene for the formation of yellow petals. The qRT–PCR analysis results were consistent with the RNA-Seq results and provided important insights into the molecular mechanisms of the flavonoid metabolic pathway in the different *Dendrobium* tissues. Our results will provide a valuable resource and a substantial basis for understanding the molecular mechanisms controlling yellow *Dendrobium* flowers, and will eventually accelerate the genetic engineering of yellow *Dendrobium* flowers.

## Figures and Tables

**Table 1:** DEGs obtained from the comparison of different tissues between the two *Dendrobium* species.

Genes	Gene ID	Upregulated	Downregulated
Flavonoid 3′-monooxygenase	LOC110113263	all five groups	
Isoflavone 7-O-glucoside-6″-O-malonyltransferase	LOC110111377	all five groups	
Flavonol synthase 6	LOC114581176	all five groups	
Flavonoid 3′-monooxygenase	LOC110115941		all five groups
Dihydroflavonol 4-reductase/flavanone 4-reductase	LOC110093920		all five groups
Shikimate O-hydroxycinnamoyltransferase	LOC110093422		2-year-old stems for all five groups
Isoflavone 7-O-glucoside-6″-O-malonyltransferase	LOC110098588	2-year-old stems for three stem tissues	
Trans-cinnamate 4-monooxygenase	LOC110101902	2-year-old stems for three stem tissues	
Isoflavone 7-O-glucoside-6″-O-malonyltransferase	LOC110109272		2-year-old stems for three stem tissues
Chalcone synthase	LOC110105073		2-year-old stems for three stem tissues
Chalcone synthase 8	LOC 110107833		2-year-old stems for three stem tissues

## Data Availability

To complete RNA-Seq data preprocessing, quality validation, and mapping to the *Dendrobium officinale* reference genome, public-domain software was used without any other custom code. These software tools and their versions are listed as follows: 1. FastQC v0.11.8 was used for quality assessment of the raw reads and the trimmed reads of RNA-sequencing data: https://www.bioinformatics.babraham.ac.uk/projects/fastqc/, accessed on 15 July 2021. 2. Trimmomatic 0.38 was used to remove adaptors and perform quality trimming: http://www.usadellab.org/cms/?page=trimmomatic, accessed on 15 July 2021. 3. MultiQC 1.0 was used to perform cross-sample quality assessment of the RNA-sequencing reads: https://multiqc.info/, accessed on 15 July 2021. 4. STAR 2.7 was used to map the cleaned RNA-Seq reads to the *Dendrobium officinale* reference genome assembly, GRCh38: https://github.com/alexdobin/STAR, accessed on 15 July 2021. 5. edgeR 3.30.3 was used to carry out trimmed mean of M values (TMM) normalization for gene expression quantification and to identify differentially expressed genes (DEGs) between sample groups: https://bioconductor.org/packages/release/bioc/html/edgeR.html, accessed on 15 July 2021.

## Supplementary Materials

The following supporting information can be downloaded at: https://www.mdpi.com/article/10.3390/ijms241311039/s1.
