# Supplementary material for: Transcriptomic and Metabolomic Analyses Reveal Differences in Flavonoid Pathway Gene Expression Profiles between Two Dendrobium Varieties during Vernalization
Source: Int J Mol Sci. 2023 Jul 3;24(13):11039. doi: 10.3390/ijms241311039 (PMC10342096; doi:10.3390/ijms241311039)
Supplement: Supplementary file 1 [file ijms-24-11039-s001.zip › Figures S1 and S2.pdf]

International Journal of
Molecular Sciences

MDPI

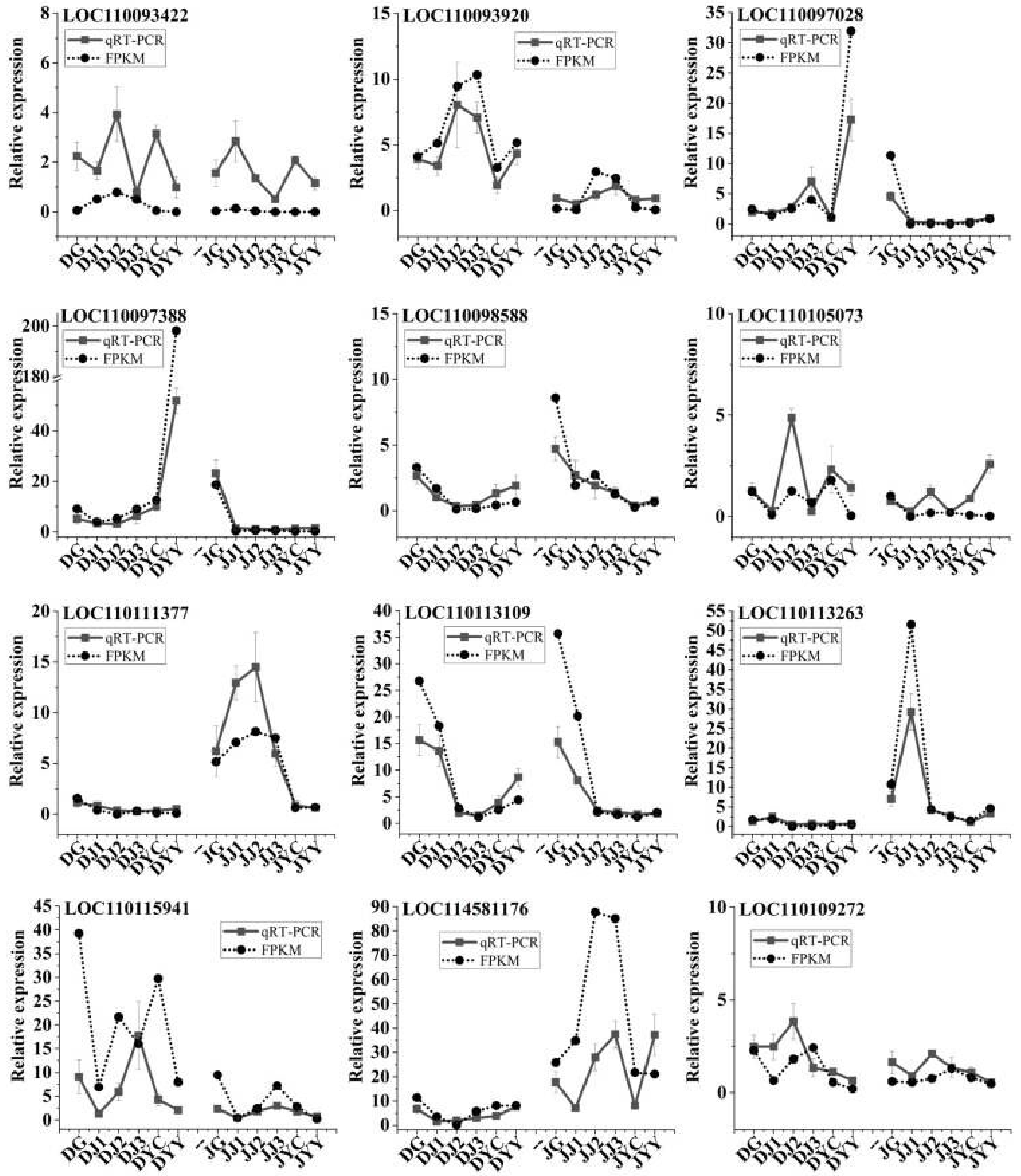

Figure S1. Verification of the expression patterns of RNA-seq results using qRT–PCR.

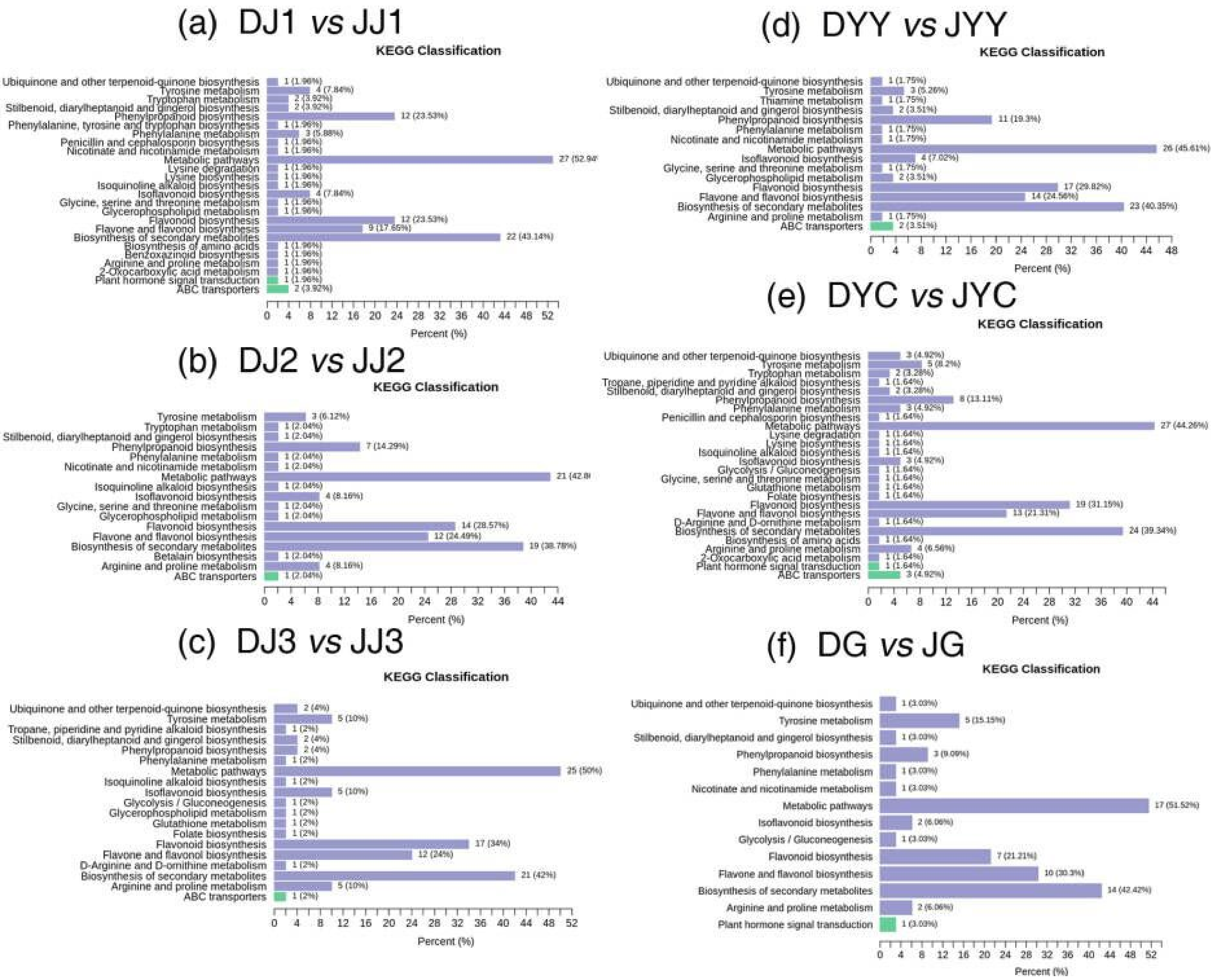

Figure S2. KEGG pathway analysis of DAMs. (a) DJ1 vs. JJI; (b) DJ2 vs. JJ2; (c) DJ3 vs. JJ3; (d) DYY vs. JYY; (e) DYC vs. JYC; (f) DG vs. JG.
