# Supplementary material for: Transcriptomic and Metabolomic Analyses Reveal Differences in Flavonoid Pathway Gene Expression Profiles between Two Dendrobium Varieties during Vernalization
Source: Int J Mol Sci. 2023 Jul 3;24(13):11039. doi: 10.3390/ijms241311039 (PMC10342096; doi:10.3390/ijms241311039)
Supplement: Supplementary file 1 [file ijms-24-11039-s001.zip › Table S1-The primer sequences.pdf]

Table S1. The primer sequences used in real-time quantitative PCR

Genes	Gene ID	Primer sequence F (5' to 3')	Primer sequence R (5' to 3')
Flavonoid 3'-monooxygenase	LOC110113263	GAACCGAGCAAAGAATCAA	TCGAAAGGGCGTGTAGTGT
Isoflavone 7-O-glucoside-6"-O-malonyltransferase	LOC110111377	ACCTCCCACTTCATACACC	CGCCGATAAACTCAGCAAC
Isoflavone 7-O-glucoside-6"-O-malonyltransferase	LOC110098588	AAACTACTTTGGGAACTGC	ATCATCGCTTCTAACACCT
Iisoflavone 7-O-glucoside-6"-O-malonyltransferase	LOC110109272	CTTATTCAACCGCCGTCTT	GTCACCGTCGGAGTTACAG
p-Coumaroyl-CoA	LOC110105073	GCAGCCTAAGTCCCGCATCA	CCGCCAGCGAAACAACCCT
Flavonol synthase 1	LOC110097028	ATAAGTATGCGATGAAGGAA	GAAAGGCTGAAACAAACAA
Flavanone 3-dioxygenase F3H2	LOC110107388	TGAAGCCAATGGATGTAAG	TTTGAAAGTCGCCAAGATA
Hydroxycinnamoyl transferase	LOC110113109	CTTGGGTTCGAGCCTTGTG	ACATTGGGTGATGTGGTGA
Flavonol synthase 6	LOC114581176	GCTCGGTATCAACGGTATG	CTTCAGTTTATTCTGTGGG
Putrescine hydroxycinnamoyl transferase 1	LOC110093422	TTCGTCCCGTACTAAAGCCAGTT	CTAACCTCCGCACAGCACTCG
Flavonoid 3'-monooxygenase	LOC110115941	CTCATATTCGCCCACTACGG	GCACGCACTTATTCCATCAC
Dihydroflavonol 4-reductase/flavanone 4-reductase	LOC110093920	GGCAGCAAGTCTAAGTGAT	GAACCGAGGAAGTTAGGAA
TBP (TATA box binding protein like)	AT1G55520.1	GGCATCCTTCTGGTATTGTCC	TACGAGCATACTTCCGAGCAG
